# Exercise-induced compensation and C-reactive protein change in adults with elevated C-reactive protein: analysis of a randomised controlled trial

**DOI:** 10.1007/s00421-026-06405-4

**Published:** 2026-08-27

**Authors:** James L. Dorling, John W. Apolzan, Diana M. Thomas, Christoph Höchsmann, Neil M. Johannsen, Robert Ross, Tim S. Church, Corby K. Martin

**Affiliations:** 1 Human Nutrition, School of Medicine, Dentistry and Nursing, College of Medical, Veterinary and Life Sciences, University of Glasgow, New Lister Building, Glasgow Royal Infirmary, 10-16 Alexandra Parade, Glasgow, G31 2ER UK; 2 Pennington Biomedical Research Center, Baton Rouge, LA USA; 3 United States Military Academy, West Point, NY USA; 4 Department of Health and Sport Sciences, TUM School of Medicine and Health, Technical University of Munich, Munich, Germany; 5 Louisiana State University, Baton Rouge, LA USA; 6 School of Kinesiology and Health Sciences, Queen’s University, Kingston, ON Canada; 7 Wondr Health, Inc., Dallas, TX USA

**Keywords:** Energy balance, Physical activity, Cardiometabolic risk, Weight regulation, Food intake, Inflammation

## Abstract

**Purpose:**

Exercise can reduce C-reactive protein (CRP), but heterogeneity exists and it is unclear whether exercise-induced weight compensation occurs or relates to CRP change. We examined whether compensation occurs in adults with elevated CRP during exercise and whether compensation and compensatory behaviours are associated with CRP change.

**Methods:**

In this 4-month randomised controlled trial, 124 sedentary adults with elevated CRP (mean ± SD: 4.3 ± 2.5 mg/L) were randomised to a control group or a supervised aerobic exercise group expending 16 kcal/kg/week. Predicted weight change was estimated, and weight compensation (weight change – predicted weight change) was calculated. CRP was measured, and energy intake and non-exercise step count were assessed using questionnaires and pedometers, respectively.

**Results:**

Weight compensation was greater in the exercise group than the control group (1.3 [95% CI, 0.6, 2.1] vs. 0.1 [95% CI, -0.6, 0.9] kg; *P* = 0.029). In response to exercise, compensation did not differ between participants with moderately higher (≤ 3 mg/L) and higher (> 3 mg/L) CRP (*P* = 0.977). Exercisers who did not compensate exhibited a 1.2 (95% CI, 0.2, 2.2) mg/L reduction in CRP versus those who did compensate (*P* = 0.016). Higher energy intake was associated with CRP change in exercisers, with each 100 kcal/day elevation in energy intake associated with a 0.1 (95% CI, 0.0, 0.2) mg/L increase in CRP (*P* = 0.022).

**Conclusion:**

Exercise-induced weight compensation occurs in adults with elevated CRP and is associated with attenuated improvements in CRP. Increased energy intake was associated with higher CRP in exercisers, suggesting that limiting increases in energy intake may enhance exercise-induced anti-inflammatory benefits.

**Clinical trials registration:**

NCT00113061.

**Supplementary Information:**

The online version contains supplementary material available at https://doi.org/10.1007/s00421-026-06405-4.

## Introduction

Cardiovascular disease (CVD) remains a leading cause of mortality in the USA (Ahmad and Anderson 2021) and the UK (Pearson-Stuttard et al. 2021). Chronic systemic inflammation contributes to the development and progression of CVD (Ferrucci and Fabbri 2018), and several markers and cytokines characterise systemic inflammation (Pepys and Hirschfield 2003). A primary marker of systemic inflammation is C-reactive protein (CRP). This is synthesised primarily by hepatocytes in response to inflammatory cytokine signalling (Pepys and Hirschfield 2003), is related to hepatic metabolism and greater visceral adipose tissue (VAT) (Brooks et al. 2010), and is independently associated with CVD risk (Koenig et al. 1999; Ridker 2001). Strategies that reduce CRP are important for lowering CVD risk and burden, and in this regard, aerobic exercise training is a promising strategy that decreases CRP regardless of age or sex, although considerable interindividual variability in CRP change suggests that unaccounted factors influence this effect (Fedewa et al. 2017).

A frequent consequence of exercise training is weight compensation, defined as actual weight loss that is less than predicted weight loss based on energy expenditure of exercise (Church et al. 2009; Martin et al. 2019). There are several behavioural and physiological factors that drive weight compensation during exercise, but an increase in energy intake is the primary mediator (Martin et al. 2019). Although exercise confers metabolic and non-metabolic benefits independent of compensation (and weight loss) (Donnelly et al. 2009), exercise-induced compensation and compensatory behaviours can attenuate favourable changes in body composition and CVD risk markers (Ross et al. 2000; Swift et al. 2016; Dorling et al. 2024). In the Inflammation and Exercise (INFLAME) study, aerobic exercise did not reduce CRP overall among participants with elevated baseline CRP, but those who lost more weight exhibited greater reductions in CRP (Church et al. 2010). This is consistent with other analyses (Fedewa et al. 2017) and suggests that compensation and compensatory behaviours may attenuate the anti-inflammatory benefits of exercise. However, no analysis has directly tested whether compensation (assessed using advanced models that predict weight change during exercise) occurs during exercise training in individuals with elevated CRP, nor to what extent change in CRP is related to exercise-induced compensation and compensatory-related mechanisms, such as energy intake and non-exercise physical activity (i.e., physical activity outside of prescribed exercise).These investigations are important as chronic mild inflammation has been associated with deleterious eating habits and increased appetite (Succurro et al. 2015; Agarwal et al. 2023), potentially suggesting that exercise-induced compensation may be greater in populations with elevated CRP. Moreover, an understanding of whether compensation and compensatory behaviours affect CRP during a supervised, controlled exercise intervention will help enhance and refine lifestyle regimens in individuals with elevated CRP who are at higher risk for CVD.

The present analysis had three principal aims. First, we tested whether aerobic exercise training causes weight compensation in individuals with elevated CRP. Second, in individuals randomised to the aerobic exercise group, we tested whether those who showed increased weight compensation (compensators) had altered change in CRP relative to those who did not compensate (non-compensators). Third, we examined whether changes in energy intake and non-exercise physical activity were associated with CRP change during exercise.

## Materials and methods

### Study design

The current manuscript is a post-hoc analysis of the INFLAME study (ClinicalTrials.gov: NCT00113061), which has been detailed previously (Thompson et al. 2008; Church et al. 2010). The study was approved by the institutional review board of the Cooper Institute, with quarterly reviews conducted by this board and a data safety and monitoring board. The INFLAME study was a 4-month randomised controlled trial in which participants with elevated CRP were randomised to either a no-exercise control group or an exercise group prescribed to expend 16 kcal/kg/week (KKW). A study statistician performed randomisation using a pseudo-random number generator and filled envelopes with treatment assignment before they were provided to health educators and participants. Blinding participants and intervention staff of the randomisation group was not possible, although the study investigators and the assessment team were blinded (Thompson et al. 2008). Recruitment began in March 2005, and data collection was completed in December 2006 once the targeted sample size was acquired. All participants provided written informed consent and procedures were in accord with the guidelines set by the Declaration of Helsinki.

### Participants

Inclusion and exclusion criteria of participants in the INFLAME study have been detailed (Thompson et al. 2008; Church et al. 2010). Briefly, participants aged 30 to 75 years who were sedentary (not exercising > 20 min on ≥ 3 days/week) and had an elevated CRP value (≥ 2.0 mg/L but < 10.0 mg/L) at the initial screening were considered for randomisation. The lower threshold for CRP screening (2.0 mg/L) was chosen as it is prevalent in people who are sedentary and is associated with elevated CVD risk (Ridker 2001); the upper CRP cutoff (10.0 mg/L) was chosen because values higher than this are frequently the result of acute infection or other inflammatory conditions (Pearson et al. 2003). Eligibility was based on the initial screening CRP value only; measurement of baseline CRP was not used to determine inclusion. Participants meeting the initial screening CRP criteria remained enrolled regardless of their baseline CRP value. More specifically, individuals with initial screening CRP < 10.0 mg/L but baseline CRP > 10.0 mg/L, and those with initial screening CRP > 2.0 mg/L but baseline CRP < 2.0 mg/L (12 controls and 12 exercisers), were retained in the primary analyses. Exclusion criteria included smoking, a body mass index (BMI) ≤ 18.5 kg/m^2^ or ≥ 40.0 kg/m^2^, a history of CVD or disorders, diabetes, (for women) current hormone therapy treatment, autoimmune or collagen vascular diseases, immunodeficiency diseases (e.g., human immunodeficiency virus), and any serious contraindications to exercising safely (e.g., musculoskeletal problems).

### Intervention

Aerobic exercise training expending 16 KKW was conducted on a treadmill and cycle ergometer within a heart rate range equivalent to 60%-80% of baseline peak oxygen uptake (V̇O_2_ peak) for 4 months (Thompson et al. 2008; Church et al. 2010). In weeks 1 and 2 of the exercise intervention, participants expended 10 KKW and 13 KKW, respectively, to acclimate to the training load; thereafter, participants expended 16 KKW. All exercise was fully supervised and monitored. Participants were weighed weekly and chose whether they wished to complete 3, 4 or 5 sessions in the forthcoming week. The energy expenditure target of each session was calculated by dividing the weekly target by the number of sessions chosen, and standardised estimations of energy expenditure rate were used to calculate the time needed to attain energy expenditure targets during sessions. Consequently, session duration varied both between and within participants. Participants were allowed to alternate between treadmill and cycle ergometer exercise to decrease injury risk and increase engagement (Thompson et al. 2008). Treadmill speed and/or grade or cycle ergometer wattage were adjusted to maintain participants’ heart rates within the target zone. Adherence to the exercise regimen was calculated as the ratio of achieved to prescribed exercise energy expenditure, expressed as a percentage.

### Outcome measures

Weight and height were measured using an electronic scale (Siemens Medical Solutions, Malvern, PA) and stadiometer, respectively, and waist circumference was measured. Further, body fat percentage was measured with dual-energy x-ray absorptiometry scans using a Hologic Bone Densitometer (Hologic, Inc., Bedford, MA), and abdominal VAT and subcutaneous adipose tissue (SAT) were examined using electron beam computed tomography (Imatron; General Electric, Milwaukee, WI) (Ross et al. 1992; Church et al. 2010). Measurement of V̇O_2_ peak was conducted using a Lode Excalibur Sport cycle ergometer (Groningen, The Netherlands), as described (Thompson et al. 2008; Church et al. 2010).

Consistent with previous work (Martin et al. 2019; Dorling et al. 2021, 2024), weight compensation was calculated as the difference between observed weight change and predicted weight change. Predicted end-of-intervention weight was derived using a validated dynamic energy balance model, which is a differential equation grounded in the first law of thermodynamics (Thomas et al. 2012, 2013). This model accounts for metabolic adaptation and potential non-linear shifts in weight during aerobic exercise training, addressing limitations associated with conventional weight prediction methods. Predicted changes in weight were based on the increase in energy expenditure due to physical activity in the exercise group (Thomas et al. 2012, 2013), while predicted weight change in the control group was assumed to be zero (Martin et al. 2019). The model does not incorporate compensatory behaviours; therefore, the discrepancy between predicted and actual weight at month 4 quantifies the extent of compensation in both groups. Dietary intake was assessed at baseline and follow-up using the Food Intake and Analysis System (FIAS; version 3.9, University of Texas Health Science Center), incorporating a semiquantitative food frequency questionnaire. Reported intake frequencies and portion sizes were converted to average daily intakes by multiplying frequency of consumption by standard portion sizes. Energy (kcal/day) and macronutrient intakes (g/day) were derived using the USDA food composition database embedded within FIAS, with food items linked to the Pyramid Servings Database to estimate food group and discretionary nutrient intake (Cleveland et al. 1997; Thompson et al. 2008). Daily non-exercise step count outside the assigned exercise sessions was monitored during waking hours of the intervention via step counters (Accusplit Eagle, Japan) and was recorded by participants daily. Average daily non-exercise step count before the intervention and during month 4 of the regimen was utilised for this analysis.

Blood samples were drawn from participants after a 10–12 h fast at baseline and follow-up. Participants did not exercise for 24 h prior to blood samples. A solid-phase, chemiluminescent immunometric assay (Immulite 2000 High-Sensitivity CRP; Diagnostic Products Corporation, Los Angeles, CA) was used to measure serum CRP (Church et al. 2010). The analytical sensitivity of the assay was 0.1 mg/L and the assay had a reportable range of 0.2–100.0 mg/L. Each participant’s set of samples were measured using the same assay kit, and as described previously (Church et al. 2010), the Pennington Biomedical Research Center clinical laboratory reported a coefficient of variation of 6.5% for CRP concentrations within the range observed in the study population.

### Statistical analysis

The current analysis assessed secondary endpoints of the INFLAME trial; therefore, power analyses were not performed for these endpoints. Participants considered for the analyses were trial completers and (for those randomised to the exercise group) had energy expenditure and baseline height and weight data (for the calculation of predicted weight change and compensation). Akin with the primary outcome paper (Church et al. 2010), we also removed participants who had CRP values more than 15.0 mg/L at baseline or follow up in the analyses, given elevated values of this kind often occur because of an acute infection or other inflammatory conditions (Rifai and Ridker 2001). All statistical analyses were performed in SPSS version 28 (SPSS Inc, Chicago, IL) and a complete case analysis was adopted.

One-way ANCOVA examined the effect of supervised aerobic exercise on weight compensation with adjustments for age, sex, and race. Similarly, one-way ANCOVAs examined between-group differences in change in energy intake, change in macronutrient intake, and change in non-exercise step count, with adjustments for age, sex, race, and baseline of the respective outcome. To identify if baseline CRP was associated with exercise-induced compensation, participants were categorised as moderately high (≤ 3.0 mg/L) and high (> 3.0 mg/L) CRP due to the link between this cut-off and CVD (Pearson et al. 2003), and two-way ANCOVA adjusted for age, sex, race and baseline values were performed to examine the interaction of baseline CRP (moderately high versus high) and randomisation group (exercise versus control) on outcomes. Like previous work (Dorling et al. 2024), we classified exercisers as compensators (weight compensation > 0 kg) and non-compensators (weight compensation ≤ 0 kg), and we examined the differences in change in CRP, weight and body composition in compensators and non-compensators during exercise via a one-way ANCOVA adjusted for age, sex, race, and respective baseline values. We also conducted an ANCOVA for CRP change adjusted for the same covariates and change in abdominal VAT. Where significant effects occurred in the ANCOVA omnibus tests, Holm-Bonferroni adjusted pairwise comparisons were used to identify where differences lay. Additionally, as sensitivity analyses, we performed multiple linear regression models to examine if weight compensation was associated with the same outcomes whilst adjusting for age, sex, race, and baseline values. Pearson correlations tested the relationship between change in CRP and change in energy intake and non-exercise step count (i.e., step count outside of prescribed exercise), and significant variables were then entered into a multiple linear regression model along with age, sex, race, and baseline CRP to assess if compensatory-related behaviours were associated with CRP change during exercise. A further multiple linear regression model was performed adjusted for the same covariates and change in abdominal VAT. Data were visually checked for approximate normality and significance for all analyses was set to α = 0.05. Unless stated otherwise, descriptive data are presented as mean ± SD and data from inferential statistical analyses are presented as estimated marginal means ± 95% CI.

## Results

### Participants

**Fig. 1:**
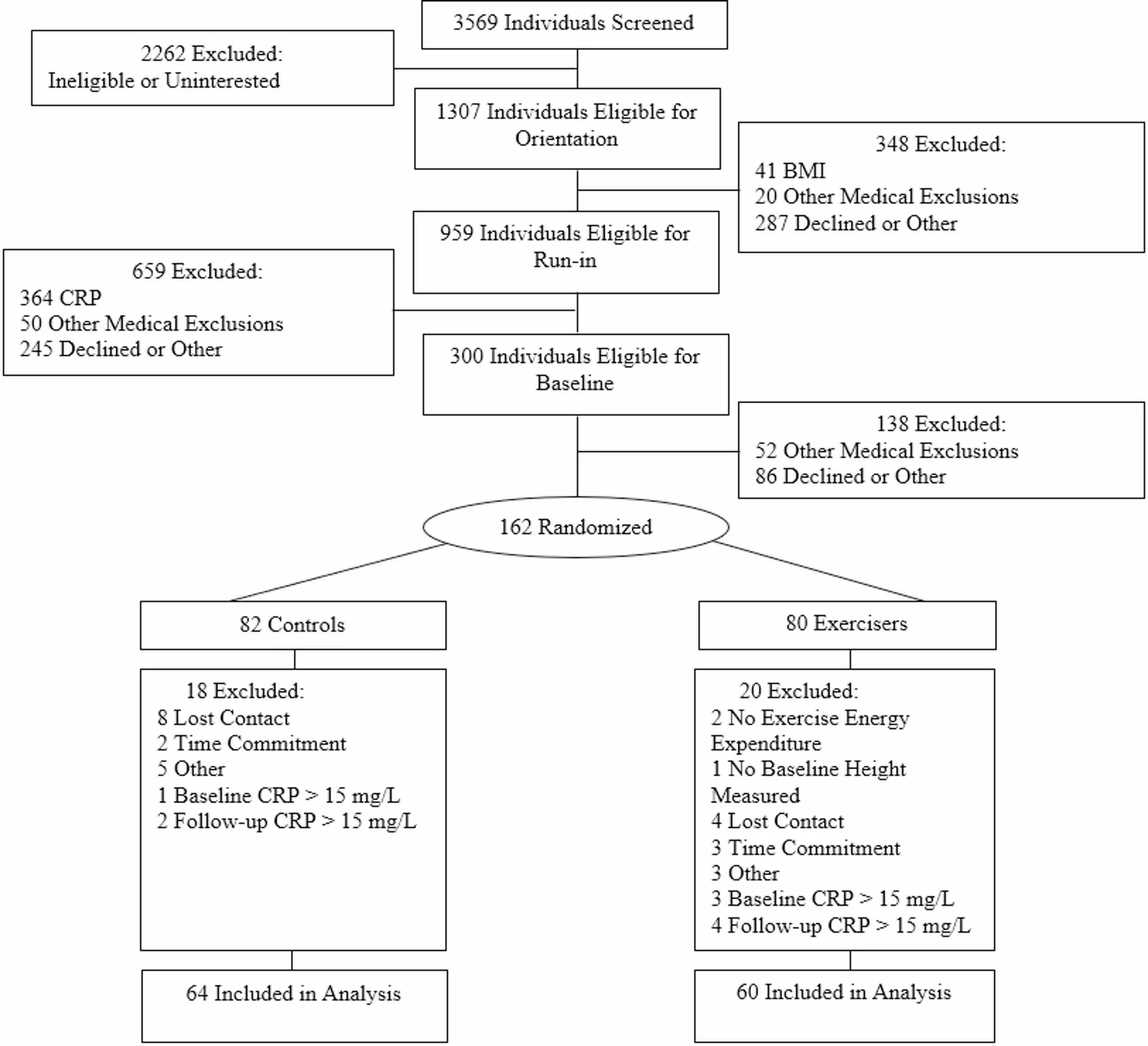
Study participant CONSORT

In total, 162 participants were randomised; of these, 124 participants (64 control, 60 exercise) were included in analyses as these completed the study, had CRP values at the baseline and follow-up assessment visits less than 15.0 mg/L, and (if randomised to the exercise group) had energy expenditure data for the calculation of compensation (Fig. 1). The mean (± SD) age, BMI, and baseline CRP of participants was 50.8 (10.8) years, 31.4 (4.1) kg/m^2^, and 4.3 (2.5) mg/L respectively, and most were female (*N* = 90; 72.6%) and white (*N* = 87; 70.2%; Table 1).

**Table 1:** Descriptive characteristics of participants who completed the trial at baseline

	Control (*N* = 64)	Exercise (*N* = 60)	All (*N* = 124)
Age (years)	50.6 (11.7)	50.9 (9.8)	50.8 (10.8)
*Sex*			
Male	22 (34.4)	12 (20.0)	34 (27.4)
Female	42 (65.6)	48 (80.0)	90 (72.6)
*Race*			
White	43 (67.2)	44 (73.3)	87 (70.2)
Black	12 (18.8)	11 (18.3)	23 (18.5)
Other	9 (14.1)	5 (8.3)	14 (11.3)
Weight (kg)	90.6 (15.8)	85.9 (14.2)	88.3 (15.2)
BMI (kg/m^2^)	31.8 (3.8)	31.0 (4.4)	31.4 (4.1)
*BMI class*			
Normal weight	2 (3.1)	3 (5.0)	5 (4.0)
Overweight	18 (28.1)	24 (40.0)	42 (33.9)
Obesity	44 (68.8)	33 (55.0)	77 (62.1)
Body fat (%)	39.0 (6.6)	40.4 (6.8)	39.7 (6.7)
Waist circumference (cm)	100.3 (12.9)	95.6 (11.6)	98.0 (12.4)
Abdominal VAT (kg)	5.5 (3.0)	4.4 (2.6)	5.0 (2.9)
Abdominal SAT (kg)	12.7 (3.6)	11.5 (4.3)	12.1 (4.0)
CRP (mg/L)	4.4 (2.6)	4.1 (2.4)	4.3 (2.5)
V̇O_2_ peak (ml/kg/min)	18.6 (5.5)	19.2 (5.4)	18.9 (5.5)
Energy intake (kcal/day)	1945 (889)	1851 (717)	1900 (808)
Protein intake (g/day)	81 (40)	79 (35)	80 (37)
Carbohydrate intake (g/day)	238 (114)	223 (86)	231 (101)
Fat intake (g/day)	76 (36)	73 (33)	75 (34)
Saturated fat intake (g/day)	25 (12)	24 (11)	25 (12)
Fiber intake (g/day)	19 (11)	19 (8)	19 (9)
Percent protein intake	17 (2)	17 (3)	17 (3)
Percent carbohydrate intake	49 (6)	49 (8)	49 (7)
Percent fat intake	35 (4)	35 (6)	35 (5)
Non-step count (steps/day)	6098 (2524)	5558 (2933)	5844 (2726)

Continuous data are mean (± SD); categorical data are number (%)

*BMI* body mass index, *CRP* c-reactive protein, *SAT* subcutaneous adipose tissue, *VAT* visceral adipose tissue, *V̇O*_2_ peak, peak oxygen uptake

### Exercise intervention data

The mean (± SD) total calories expended by the exercise group during supervised training was 17,996 (5,329) kcals, with a mean energy expenditure of 1,184 (225) kcal/week. Participants were prescribed 18,389 (5,450) kcals in total, and the adherence of participants in the exercise group was 98.2% (5.9%), indicating excellent adherence.

### Compensation and energy intake change

Compensation and change scores in the exercise and control groups are displayed in Table 2. Akin to the primary outcome article (Church et al. 2010), exercise training did not affect waist circumference, abdominal VAT, abdominal SAT or CRP compared to control (all *P* ≥ 0.063), but training did decrease body fat and increase V̇O_2_ peak (*P* ≤ 0.021). Our energy balance model predicted that the exercise group would display − 2.1 kg (95% CI, -2.2, -1.9) weight change compared to control (*P* < 0.001), but actual weight change was not different between groups (between-group difference: -0.9 kg [95% CI, -1.9, 0.2]; *P* = 0.116). There was significant weight compensation displayed by the exercise group (1.3 kg [95% CI, 0.6, 2.1]), which was greater than the control group (0.1 kg [95% CI, -0.6, 0.9]; *P* = 0.029); yet there were no differences in energy or macronutrient intake or non-exercise step count between groups (all *P* ≥ 0.247; Table 2). The changes in weight, compensation, body composition, CRP, and step count were similar during exercise in individuals with moderately high (≤ 3.0 mg/L) and high CRP (> 3.0 mg/L) at baseline, as shown in Table 3 (all *P* for interactions ≥ 0.238). There was a significant interaction between study group and baseline CRP levels for change in percent fat intake (*P* for interaction = 0.009), but following Holm-Bonferroni adjusted pairwise comparisons, there was no significant differences between the exercise and control groups when individuals with moderately high (between-group difference: 2% [95% CI, 0, 5]) and high (between-group difference: -2% [95% CI, -4, 0]) baseline CRP concentrations were analysed separately (*P* ≥ 0.071). A main effect of CRP category showed that the individuals with high baseline CRP exhibited a -0.5 kg (95 CI, -0.9, 0.0) change in abdominal SAT compared to individuals with moderately high baseline CRP (*P =* 0.044), but there was no main effect of exercise (*P* = 0.128) and no significant interaction between study group and baseline CRP category (*P* = 0.965).

**Table 2:** Compensation and change in outcome variables from baseline to month 4 by group

	Control (*N* = 64)	Exercise (*N* = 60)	Between-group difference	*P* value
Actual weight (kg)	0.1 (-0.6, 0.9)	-0.7 (-1.5, 0.0)	-0.9 (-1.9, 0.2)	0.116
Predicted weight (kg)	0.0 (-0.1, 0.1)	-2.1 (-2.2, -2.0)	-2.1 (-2.2, -1.9)	< 0.001^*^
Compensation (kg)^a^	0.1 (-0.6, 0.9)	1.3 (0.6, 2.1)	1.2 (0.1, 2.3)	0.029^*^
Actual percent weight (%)	0.1 (-0.7, 0.9)	-0.9 (-1.8, 0.0)	-1.0 (-2.2, 0.2)	0.107
Body fat (%)	0.1 (-0.3, 0.5)	-0.6 (-1.0, -0.2)	-0.7 (-1.2, -0.1)	0.021^*^
Waist circumference (cm)	-0.1 (-1.3, 1.1)	-1.3 (-2.5, 0.0)	-1.2 (-2.9, 0.6)	0.183
Abdominal VAT (kg)	0.0 (-0.2, 0.1)	-0.3 (-0.4, -0.1)	-0.2 (-0.4, 0.0)	0.063
Abdominal SAT (kg)	0.0 (-0.3, 0.3)	-0.3 (-0.7, 0.0)	-0.3 (-0.8, 0.1)	0.132
CRP (mg/L)	0.3 (-0.2, 0.7)	0.0 (-0.5, 0.4)	-0.3 (-1.0, 0.4)	0.390
V̇O_2_ peak (ml/kg/min)	-0.1 (-0.7, 0.5)	2.4 (1.8, 3.1)	2.6 (1.7, 3.5)	< 0.001^*^
Energy intake (kcal/day)	-118 (-246, 10)	-114 (-244, 16)	4 (-180, 188)	0.965
Protein intake (g/day)	-5 (-11, 1)	-3 (-9, 3)	2 (-7, 10)	0.661
Carbohydrate intake (g/day)	-9 (-26, 8)	-8 (-25, 9)	1 (-23, 26)	0.914
Fat intake (g/day)	-7 (-12, -1)	-7 (-13, -1)	-1 (-9, 8)	0.902
Saturated fat intake (g/day)	-2 (-4, 0)	-3 (-5, -1)	-1 (-3, 2)	0.679
Fiber intake (g/day)	-1 (-2, 1)	1 (-1, 3)	1 (-1, 4)	0.247
Percent protein intake	0 (-1, 1)	1 (0, 1)	1 (0, 1)	0.263
Percent carbohydrate intake	1 (-1, 2)	1 (-1, 3)	0 (-2, 3)	0.737
Percent fat intake	-1 (-2, 0)	-1 (-2, 0)	-1 (-2, 1)	0.553
Non-step count (steps/day)	931 (299, 1562)	1018 (368, 1668)	88 (-825, 1001)	0.849

Data are estimated marginal means (95% CI) adjusted for age, sex, race, and (if appropriate) baseline variables

*CRP* c-reactive protein, *SAT* subcutaneous adipose tissue, *VAT* visceral adipose tissue, *V̇O*_2_ peak, peak oxygen uptake

^*^Significant between-group difference from ANCOVA (*P* < 0.05)

^a^Compensation (kg) is actual weight change (kg) minus predicted weight change (kg)

**Table 3:** Compensation and change in outcome variables from baseline to month 4 by group (exercise versus control) in those with moderately high (≤ 3 mg/L) and high (> 3 mg/L) CRP at baseline

	Moderately high baseline CRP (≤ 3 mg/L)		High baseline CRP (> 3 mg/L)		Study group *P*	Baseline CRP category *P*	Study group x baseline CRP *P*
	Control (*N* = 25)	Exercise (*N* = 24)	Control (*N* = 39)	Exercise (*N* = 36)			
Actual weight (kg)	0.3 (-0.9, 1.6)	-0.4 (-1.6, 0.9)	0.0 (-0.9, 1.0)	-0.9 (-1.9, 0.1)	0.140	0.476	0.824
Predicted weight (kg)	0.1 (-0.1, 0.2)	-2.0 (-2.2, -1.8)	0.0 (-0.2, 0.1)	-2.1 (-2.3, -2.0)	< 0.001^*^	0.169	0.550
Compensation (kg)^a^	0.3 (-1.0, 1.5)	1.5 (0.2, 2.7)	0.1 (-0.9, 1.0)	1.3 (0.3, 2.3)	0.038^*^	0.717	0.977
Actual percent weight (%)	0.3 (-1.1, 1.7)	-0.4 (-1.9, 1.0)	0.0 (-1.1, 1.1)	-1.2 (-2.4, -0.1)	0.136	0.409	0.741
Body fat (%)	0.4 (-0.3, 1.0)	-0.8 (-1.4, -0.1)	-0.1 (-0.6, 0.4)	-0.5 (-1.0, 0.0)	0.012^*^	0.796	0.238
Waist circumference (cm)	-0.4 (-2.4, 1.6)	-0.7 (-2.8, 1.3)	0.0 (-1.5, 1.6)	-1.6 (-3.2, 0.1)	0.273	0.830	0.489
Abdominal VAT (kg)	-0.1 (-0.4, 0.1)	-0.4 (-0.6, -0.1)	0.0 (-0.2, 0.2)	-0.2 (-0.4, 0.0)	0.065	0.244	0.740
Abdominal SAT (kg)	0.3 (-0.2, 0.8)	-0.1 (-0.6, 0.5)	-0.2 (-0.6, 0.2)	-0.5 (-0.9, -0.1)	0.128	0.044^*^	0.965
CRP (mg/L)	0.6 (-0.3, 1.5)	0.5 (-0.4, 1.3)	0.0 (-0.6, 0.7)	-0.4 (-1.0, 0.3)	0.447	0.164	0.731
V̇O_2_ peak (ml/kg/min)	-0.4 (-1.4, 0.6)	2.8 (1.8, 3.8)	0.0 (-0.8, 0.8)	2.2 (1.4, 3.0)	< 0.001^*^	0.866	0.280
Energy intake (kcal/day)	-171 (-387, 44)	-253 (-457, -49)	-87 (-250, 76)	-15 (-184, 154)	0.961	0.101	0.425
Protein intake (g/day)	-8 (-18, 2)	-10 (-19, 0)	-4 (-11, 4)	1 (-7, 9)	0.723	0.106	0.460
Carbohydrate intake (g/day)	-11 (-40, 17)	-32 (-58, -5)	-8 (-29, 13)	9 (-13, 31)	0.883	0.088	0.145
Fat intake (g/day)	-11 (-21, -1)	-10 (-19, 0)	-4 (-11, 4)	-5 (-13, 3)	0.963	0.198	0.744
Saturated fat intake (g/day)	-4 (-7, -1)	-4 (-7, -1)	-1 (-4, 1)	-2 (-4, 0)	0.798	0.140	0.843
Fiber intake (g/day)	-1 (-4, 2)	-1 (-3, 2)	0 (-2, 2)	2 (0, 4)	0.293	0.185	0.532
Percent protein intake	0 (-1, 1)	1 (0, 2)	0 (-1, 1)	0 (0, 1)	0.267	0.890	0.847
Percent carbohydrate intake	2 (-1, 4)	-1 (-3, 2)	0 (-2, 2)	2 (0, 4)	0.924	0.691	0.072
Percent fat intake	-2 (-4, 0)	0 (-2, 2)	0 (-1, 2)	-2 (-4, -1)	0.954	0.784	0.009^*^
Non-exercise step count (steps/day)	1072 (25, 2119)	507 (-526, 1539)	830 (13, 1647)	1371 (508, 2234)	0.979	0.528	0.248

Data are estimated marginal means (95% CI) adjusted for age, sex, race, and (if appropriate) baseline variables

Abbreviations: CRP, c-reactive protein; SAT, subcutaneous adipose tissue; VAT, visceral adipose tissue; V̇O_2_ peak, peak oxygen uptake

^*^Significant effect from ANCOVA (*P* < 0.05)

^a^Compensation (kg) is actual weight change (kg) minus predicted weight change (kg)

Of those 60 participants who were randomised to exercise, 45 participants (75.0%) lost less weight than expected and were classed as compensators, whereas 15 participants (25.0%) were non-compensators (i.e., lost weight equal to that or more than expected). There was a decrease in CRP levels in non-compensators (-1.0 mg/L [95% CI: -1.8, -0.1]) compared to compensators (0.3 mg/L [95% CI: -0.2, 0.7]; *P* = 0.016; Table 4). Compensators also displayed an increase in waist circumference, body fat, abdominal VAT, and abdominal SAT when compared to non-compensators (*P* ≤ 0.029). In sensitivity analyses, multiple linear regression analyses adjusted for sex, age, race, and baseline values indicated that an increase in weight compensation was associated with an increase in CRP, waist circumference, body fat, abdominal VAT, and abdominal SAT (*P* ≤ 0.014; Supplementary Table 1). Further, non-compensators still displayed a decrease in CRP compared to compensators when change in VAT was included as an additional covariate (between-group difference: 1.2 mg/L [95% CI, 0.1, 2.4]; *P* = 0.034).

**Table 4:** Change in CRP, weight, body fat, waist circumference, abdominal VAT, and abdominal SAT from baseline to month 4 in compensators and non-compensators

	Compensators (*N* = 45)	Non-compensators (*N* = 15)	Between-group difference	*P* value
CRP (mg/L)	0.3 (-0.2, 0.7)	-1.0 (-1.8, -0.1)	1.2 (0.2, 2.2)	0.016^*^
Actual weight (kg)	0.5 (-0.2, 1.2)	-4.5 (-5.8, -3.3)	5.0 (3.5, 6.5)	< 0.001^*^
Actual percent weight (%)	0.5 (-0.3, 1.4)	-5.4 (-6.9, -4.0)	6.0 (4.2, 7.7)	< 0.001^*^
Body fat (%)	-0.1 (-0.6, 0.4)	-2.1 (-2.9, -1.2)	1.9 (0.9, 2.9)	< 0.001^*^
Waist circumference (cm)	-0.2 (-1.7, 1.3)	-4.0 (-6.5, -1.4)	3.7 (0.8, 6.7)	0.015^*^
Abdominal VAT (kg)	-0.1 (-0.3, 0.1)	-0.6 (-0.9, -0.3)	0.5 (0.2, 0.8)	0.005^*^
Abdominal SAT (kg)	-0.1 (-0.5, 0.3)	-1.0 (-1.7, -0.3)	0.9 (0.1, 1.7)	0.029^*^

Data are estimated marginal means (95% CI) adjusted for age, sex, race, and baseline variables

* CRP* c-reactive protein, *SAT* subcutaneous adipose tissue, *VAT* visceral adipose tissue

*Significant effect from ANCOVA (*P* < 0.05)

**Fig. 2:**
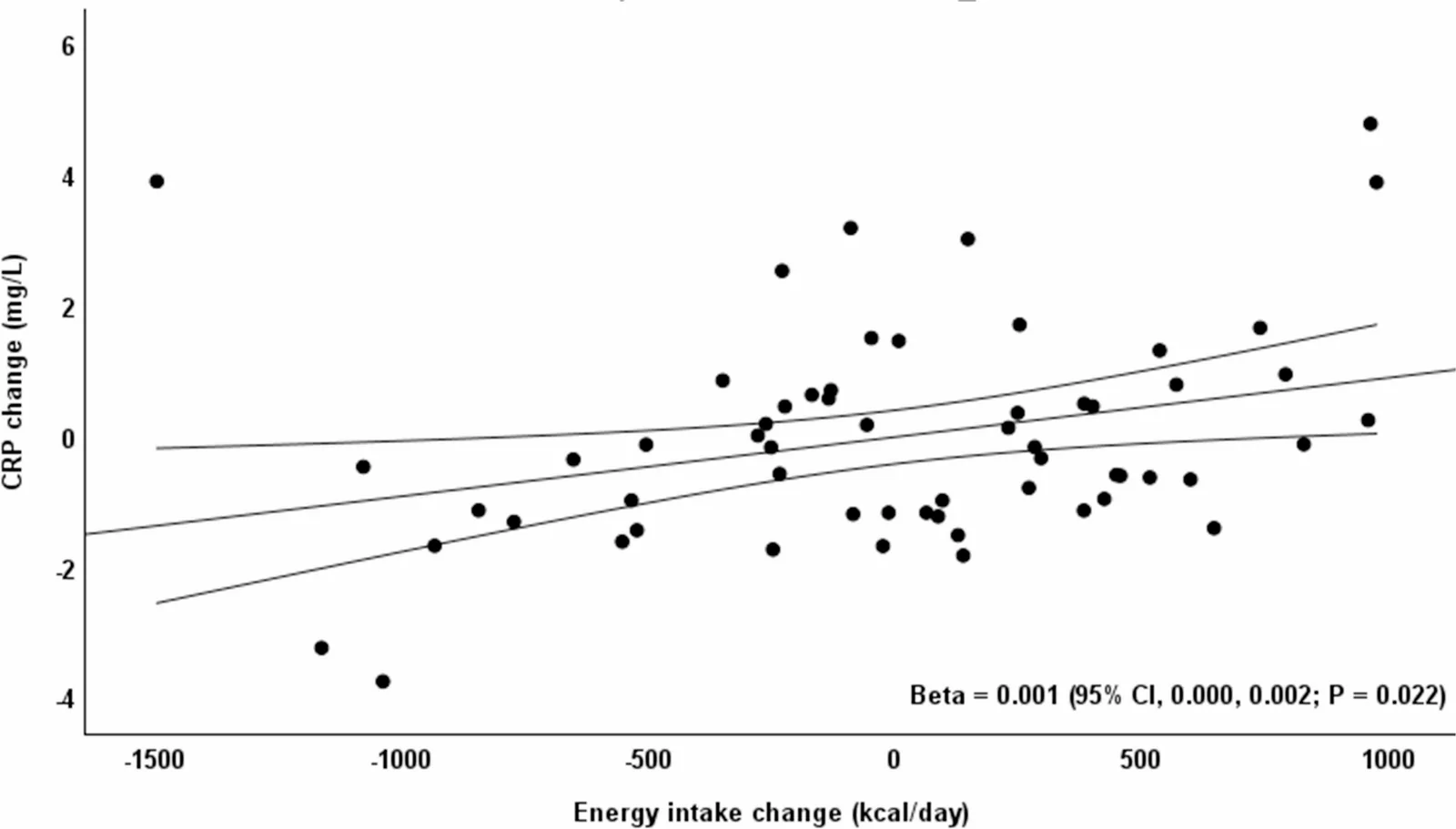
Association between change in energy intake and change in CRP in exercisers. The plot represents the relationship between change in energy intake and change in CRP after adjustment for age, sex, race, and baseline CRP, derived from a multiple linear regression model. The solid line depicts the line of best fit from linear regression with 95% confidence intervals for the mean. Abbreviations: CRP, c-reactive protein

Unadjusted Pearson correlations revealed that change in CRP was positively related to change in energy intake (*r* = 0.298; *P* = 0.022) but not associated with change in step count (*r* = -0.032; *P* = 0.821). Multiple linear regression showed a significant relationship between change in energy intake and change in CRP concentrations after adjustment of covariates, with each 100 kcal/day increase in energy intake associated with a 0.1 mg/L (95% CI, 0.0, 0.2) increase in CRP during training (*P* = 0.022; Fig. 2). Change in energy intake remained associated with change in CRP when change in VAT was an additional covariate in the multiple linear regression analysis (Beta = 0.001 [95% CI, 0.000, 0.002]; *P* = 0.026).

## Discussion

The aim of the current analysis was, in a sample of individuals with elevated CRP, to examine the impact of aerobic exercise on compensation, and to examine the association between weight compensation and compensatory-related mechanisms and change in CRP during training. Our findings suggest that exercise led to weight compensation, with no differences displayed in compensation or compensatory mechanisms in individuals with moderately high or high CRP in response to aerobic exercise training. The results indicate, moreover, that those who displayed positive weight compensation (i.e., lost less weight than expected) showed an attenuated reduction in CRP compared to non-compensators during training, and that change in energy intake was positively associated with change in CRP during exercise.

Compensation in response to aerobic exercise training occurs in individuals with overweight and obesity, and it occurs primarily because of elevations in energy intake (Martin et al. 2019; Dorling et al. 2021). Chronically elevated CRP has been associated with deleterious eating behaviours like elevated appetite, food cravings (Agarwal et al. 2023), and binge eating (Succurro et al. 2015), suggesting that compensatory responses to exercise may be greater in populations with elevated CRP. Our analysis is the first to show that exercise-induced compensation occurs in individuals who have elevated CRP using advanced weight prediction models. We also showed individuals with moderately high (≤ 3.0 mg/L) and high (> 3.0 mg/L) CRP levels displayed similar levels of compensation during exercise training, suggesting CRP levels do not affect the degree of compensation during aerobic exercise. However, the exercise-induced compensation notwithstanding, it is unclear what mechanisms are mediating the compensation shown by exercisers in the INFLAME trial, because we did not find any differences in energy intake or non-exercise step count between the exercise and control groups, as well as groups who had moderately high and high CRP at baseline. We did observe an interaction between baseline CRP levels (moderately high versus high baseline CRP) and trial condition (exercise versus control) on percent intake from fat, but post-hoc comparisons indicated no significant variations between exercise and control groups when individuals with baseline CRP < 3 mg/L and ≥ 3 mg/L were analysed separately. More specifically, percent fat intake change differed by only 2% in exercise and control groups in both those with moderately high and high baseline CRP. It is thus unlikely that any variations in percent fat intake impacted CRP concentrations in response to exercise in our study, particularly in light of evidence indicating that the impact of percent fat intake on CRP is equivocal (Pickworth et al. 2019). Further work is needed to precisely identify the energy intake and macronutrient intake changes in individuals with elevated CRP in response to aerobic exercise training. Martin et al. (2019) used sophisticated measures like doubly labelled water for 14 days to show that energy intake is the principal driver of compensation during exercise. Measures such as these would have better elucidated the effect of exercise on food intake in the INFLAME trial.

Meta-analyses have revealed that aerobic exercise decreases CRP levels, with greater CRP improvements shown in those who lose more weight (Fedewa et al. 2017). In the INFLAME trial, exercise did not improve CRP relative to control, but individuals with increased weight loss displayed a greater decrease in CRP (Church et al. 2010). We found that individuals in this analysis who showed negative or zero compensation (i.e., lost weight equal to or more than predicted) decreased their CRP concentrations by an average of 1.2 mg/L compared to compensators. We can tentatively estimate, using previous evidence (Kaptoge et al. 2010), that this reduction in CRP among non-compensators compared with compensators could correspond to an estimated lower relative risk of coronary heart disease of up to 14%, depending on absolute CRP concentrations. These findings support the results of the primary manuscript (Church et al. 2010) and research demonstrating that weight loss influences CRP reductions during exercise (Fedewa et al. 2017). In addition, we found that the increase in energy intake was associated with elevations in CRP during exercise, including after the adjustment of change in VAT which is linked to CRP levels (Brooks et al. 2010) and energy intake (Merritt et al. 2024). This indicates that, independent of VAT change, an increase in energy intake is a key mechanism attenuating exercise-induced improvements in CRP. Although numerous studies have demonstrated that reductions in energy intake can suppress CRP (Imayama et al. 2012; Ravussin et al. 2015), that elevations in energy intake were related to increased CRP during exercise is novel and has important implications. Indeed, compensatory elevations in energy intake could be targeted to optimise to CRP reductions in individuals who adopt exercise as a method of decreasing their risk for CVD and other diseases, such as type 2 diabetes (Swift et al. 2012). Research is needed to identify effective methods that suppress compensatory elevations in energy intake and decrease CRP during aerobic exercise training, but simultaneous interventions that help manage appetite, promote portion size control (Dorling et al. 2022), and implement meal plans (Rickman et al. 2011) could be fruitful. Additionally, while our findings show that exercise alone improves fitness (which likely enhances health in individuals with elevated inflammation (Laukkanen et al. 2022), our results may imply that CRP improvements may be greater in response to lifestyle interventions targeting both diet and exercise.

Although increased energy intake is a principal mechanism driving compensation during aerobic exercise, reductions in non-exercise energy expenditure via decreases in non-exercise steps may also play a role. In our analysis, change in non-exercise step count was not associated with change in CRP during exercise training. Some have demonstrated that the addition of non-exercise physical activity interventions during an aerobic exercise regimen enhances reductions in weight and adiposity (Swift et al. 2021), yet our work suggests that increasing non-exercise physical activity is ineffective at enhancing CRP reductions during aerobic exercise training in individuals with elevated CRP. We should, however, acknowledge that studies are needed with more sophisticated measures of non-exercise physical activity that capture both volume and intensity of activity, such as triaxial accelerometery (Brage et al. 2015). Further, work is required to test whether any alterations in other factors modulating energy balance are associated with changes in CRP in cohorts akin to ours. This includes studies that assess non-exercise energy expenditure (e.g., resting and sleeping metabolic rate and physical activity energy expenditure), given that changes in non-exercise energy expenditure can occur during aerobic exercise in individuals who are sedentary (Broskey et al. 2021). It also includes research that examines psychological and behavioural variables related to both CRP and energy intake, such as stress and anxiety (Kennedy and Niedzwiedz 2022).

A strength of the present analysis is that it utilises data from a randomised controlled trial where exercise was supervised and adherence was excellent. That this was a post-hoc analysis is a limitation. Furthermore, although we excluded individuals with serious medical conditions (e.g., conditions likely to interfere inflammatory status) and those with CRP values < 15.0 mg/L, we cannot completely discount variations in hepatic metabolic status (Brooks et al. 2010) and acute inflammatory changes (Pepys and Hirschfield 2003) influencing circulating CRP concentrations. It is additionally a limitation that the study was only 4 months and comprised a greater proportion of females and individuals who were white. Our analysis would also have benefited from complete assessments of other pro-inflammatory cytokines and markers of metabolic endotoxemia, such as lipopolysaccharide, to supply stronger inferences regarding changes to whole-body systemic inflammation and enhance mechanistic interpretations. Finally, we should acknowledge that there are limitations with self-reported food intake questionnaires (Dhurandhar et al. 2015) and pedometers (Bassett et al. 2017) when assessing free-living energy intake and physical activity, respectively, which may have decreased sensitivity in discerning between-group differences in these outcomes, and which means further studies using additional measures are needed to verify findings. Nonetheless, the sensitivity of our analysis was enhanced because of the measurement of within-participant changes in energy intake before and after the regimen (de Castro 2006), potentially allowing us to detect the association between energy intake change and CRP change in exercisers. Likewise, given our strict inclusion/exclusion criteria, we feel our findings have direct relevance for populations who are at increased risk for CVD (Thompson et al. 2008; Church et al. 2010).

In conclusion, aerobic exercise training leads to compensation in individuals with elevated CRP, although the mechanisms driving this response are unclear. Individuals who compensated during exercise exhibited attenuated improvements in CRP compared to those who did not compensate, and elevations in energy intake during training were associated with increased CRP concentrations. Our findings show how behavioural strategies that decrease compensatory elevations in energy intake during aerobic exercise may reduce CRP and optimise long-term disease risk reduction in at-risk populations.

## Supplementary Information

Below is the link to the electronic supplementary material.

Supplementary Material 1

## Data Availability

Some or all datasets generated during and/or analysed during the current study are not publicly available but are available from the corresponding author on reasonable request. The corresponding author holds the analytical code and is willing to share on request.
