# Supplementary material for: Exercise-induced compensation and C-reactive protein change in adults with elevated C-reactive protein: analysis of a randomised controlled trial
Source: Eur J Appl Physiol. 2026 Aug 27;126(11):6329–39. doi: 10.1007/s00421-026-06405-4 (PMC13751706; doi:10.1007/s00421-026-06405-4)
Supplement: Supplementary file 1 [file 421_2026_6405_MOESM1_ESM.docx]

**Supplementary Information**

Exercise-induced compensation and C-reactive protein change in adults with elevated C-reactive protein: analysis of a randomised controlled trial

James L. Dorling^1^, John W. Apolzan^2^, Diana M. Thomas^3^, Christoph Höchsmann^4^, Neil Johannsen^2,5^, Robert Ross^6^, Tim S. Church^2^, Corby K. Martin^2^

^1^Human Nutrition, School of Medicine, Dentistry and Nursing, College of Medical, Veterinary and Life Sciences, University of Glasgow, Glasgow, United Kingdom.

^2^Pennington Biomedical Research Center, Baton Rouge, LA, USA.

^3^United States Military Academy, West Point, NY, USA.

^4^Department of Health and Sport Sciences, TUM School of Medicine and Health, Technical University of Munich, Munich, Germany.

^5^Louisiana State University, Baton Rouge, LA, USA.

^6^School of Kinesiology and Health Sciences, Queen’s University, Kingston, ON, Canada.

Supplementary Table 1. Association between weight compensation and change in CRP, weight, body fat, and waist circumference.

|  |  | B | 95% CI | *P* |
| --- | --- | --- | --- | --- |
| Weight compensation (kg)^a, b^ |  |  |  |  |
|  | CRP (mg/L) | 0.2 | 0.0, 0.3 | 0.014 |
|  | Actual weight (kg) | 1.0 | 0.9, 1.0 | <0.001 |
|  | Actual percent weight (%) | 1.1 | 1.1, 1.2 | <0.001 |
|  | Body fat (%) | 0.4 | 0.3, 0.5 | <0.001 |
|  | Waist circumference (cm) | 0.6 | 0.2, 1.0 | 0.005 |
|  | Abdominal VAT (kg) | 0.1 | 0.0, 0.1 | <0.001 |
|  | Abdominal SAT (kg) | 0.2 | 0.1, 0.3 | <0.001 |

Values are unstandardized regression coefficients with 95% confidence intervals (CI).

Abbreviations: CRP, C-reactive protein; SAT, subcutaneous adipose tissue; VAT, visceral adipose tissue.

^a^Compensation (kg) is actual weight change (kg) minus predicted weight change (kg).

^b^All models were adjusted for age, sex, race, and the baseline value of the respective dependent variable.
